# Insights into the Metabolome of the Cyanobacterium *Leibleinia gracilis* from the Lagoon of Tahiti and First Inspection of Its Variability

**DOI:** 10.3390/metabo10050215

**Published:** 2020-05-24

**Authors:** Hiren Solanki, Manon Pierdet, Olivier P. Thomas, Mayalen Zubia

**Affiliations:** 1 Marine Biodiscovery, School of Chemistry and Ryan Institute, National University of Ireland Galway, University Road, H91 TK33 Galway, Ireland; H.SOLANKI1@nuigalway.ie; 2 University of French Polynesia, UMR Ecosystèmes Insulaires Océaniens, LabEx CORAIL, BP6570, Faa’a, 98702 Tahiti, French Polynesia; manon.pierdet@gmail.com

**Keywords:** cyanobacteria, *Leibleinia*, comparative metabolomic, molecular network, Tahiti, fatty acids

## Abstract

Cyanobacteria are known to produce a large diversity of specialized metabolites that can cause severe (eco)toxicological effects. In the lagoon of Tahiti, the benthic cyanobacterium *Leibleinia gracilis* is commonly found overgrowing the proliferative macroalga *Turbinaria ornata* or dead branching corals. The specialized metabolome of the cyanobacterium *L. gracilis* was therefore investigated together with its variability on both substrates and changes in environmental parameters. For the study of the metabolome variability, replicates of *L. gracilis* were collected in the same location of the lagoon of Tahiti before and after a raining event, both on dead corals and on *T. ornata*. The variability in the metabolome was inferred from a comparative non-targeted metabolomic using high resolution mass spectrometry (MS) data and a molecular network analysis built through MS/MS analyses. Oxidized fatty acid derivatives including the unusual 11-oxopalmitelaidic acid were found as major constituents of the specialized metabolome of this species. Significant variations in the metabolome of the cyanobacteria were observed, being more important with a change in environmental factors. Erucamide was found to be the main chemical marker highly present when the cyanobacterium grows on the macroalga. This study highlights the importance of combined approaches in metabolomics and molecular networks to inspect the variability in the metabolome of cyanobacteria with applications for ecological questions.

## 1. Introduction

Cyanobacteria represent old and essential constituents of aquatic ecosystems, where they play key ecological roles as primary producers [1], fixing carbon (oxyphototrophy) and nitrogen (diazotrophy), and therefore serve as a support for numerous planktonic and benthic heterotrophs [2]. In coral reefs of the Pacific Ocean, and under normal oligotrophic conditions, some cyanobacterial populations are observed on dispersed lifeless or living substrates, forming colonies and local benthic blooms. In recent years, larger blooms of benthic cyanobacteria have occurred with increasing frequency in coral reefs and tropical lagoons, probably in response to natural and man-made environmental disturbances [3,4]. Blooms of cyanobacteria have been shown to impair coral recruitment [5,6], and some cyanobacteria of the genera *Geitlerinema*, *Leptolyngbya*, *Phormidium*, and *Pseudoscillatoria* were also identified as pathogens in coral “Black Band Disease” [7,8,9].

Some negative impacts of blooms of cyanobacteria have been related to the production of bioactive metabolites. Species of the genera *Lyngbya* or *Moorea* were for instance identified as outstanding producers of bioactive metabolites with over 800 compounds identified so far [10,11]. These metabolites can be toxic to humans, but they can also have negative impacts on other interacting species. In some cases, allelopathy was proposed to explain the effect of some metabolites on other organisms of the coral reefs therefore shaping the benthic community of these ecosystems [12]. A well-known function of cyanobacterial metabolites is to provide chemical protection from abundant and diverse herbivores [13,14,15]. Then, selective grazing of macroalgae by herbivorous fishes can remove potential competitors and favor the establishment of unpalatable, benthic cyanobacteria in reef habitats [16,17]. Recently, Morrow et al. [18] demonstrated that allelochemicals of the cyanobacteria *Lyngbya majuscula* and *Lyngbya polychroa* affect the composition and abundance of coral-associated microorganisms that control host responses and adaptations to environmental change, including susceptibility to bacterial diseases. 

French Polynesia is not an exception in the occurrence of cyanobacteria blooms, which are likely to increase with global change and anthropogenic pressures on these fragile marine ecosystems [6]. Only a limited number of studies have been reported on the diversity, and blooms of marine cyanobacteria populations in this part of the tropical Pacific Ocean [2,19,20,21,22,23,24,25]. Chemical studies performed on benthic cyanobacteria present in this area are restricted to both dominant species *Lyngbya majuscula* and *Anabaena torulosa* present in the Moorea lagoon [26,27,28,29]. The present study focuses on the less studied cyanobacterium *Leibleinia gracilis* (Rabenhorst ex Gomont) Anagnostidis and Komáerek, formerly known as *Lyngbya gracilis* Rabenhorst ex Gomont and then *Phormidium gracile* (Rabenhorst ex Gomont) Anagnostidis, which are now largely distributed in the lagoon of Tahiti. While the first chemical investigations on this species reported the presence of a macrolide debromoaplysiatoxin [30] and the alkylpyridine louludinium chloride [31] from *Lyngbya gracilis* collected in the Marshall Islands and Hawaii, respectively, the only chemical study performed on *Phormidium gracile* provided the cyclic depsipeptide hoiamide A from a consortium of species collected in Papua New Guinea [32]. Inconsistencies in the taxonomy of this species prompted us to first undertake a full chemical study of the strain present in the lagoon of Tahiti. We then wanted to study the variability of the metabolome of this species when changing some environmental factors.

Metabolomic approaches have become highly relevant to study the variability in the metabolome of marine organisms, especially when they are in interaction [33,34,35]. The increasing sensitivity and resolution of analytical techniques such as liquid chromatography (LC)-MS allow for the observation of a large array of metabolites representing key phenotypic characters of the studied organisms. Therefore, this approach gives highly relevant insights into the fitness of the organisms and the variability of their metabolome. Thus, the variability of the metabolome of this species was then investigated before and after a rainy event affecting the surrounding environmental conditions, but also with a change in substrate. *Leibleinia gracilis* is indeed observed as epiphyte of different substrates such as dead coral, rubbles, and various macroalgae of the genera *Halimeda*, *Galaxaura*, *Dictyota*, and *Turbinaria*. *Turbinaria ornata* (Turner) J. Agardh is a brown alga species (Fucales, Sargassaceae) widely distributed in tropical and subtropical areas of the Pacific and Indian Oceans. The alga forms monopodial axes attached to the substratum by stilt-like haptera, is 3–20 cm tall and 5 cm broad, coarse, and firm. Over the past three decades, *T. ornata* has bloomed in French Polynesia, accompanied by reef degradation [36,37]. These changes have very likely decreased coral recruitment, and *T. ornata* is now dominant in several parts of the reefs in Tahiti Island. *Turbinaria ornata* has been recently reported as a proliferative brown alga in the lagoon of Tahiti, and we wanted to inspect the possible change in the metabolome of the cyanobacterium commonly found as epiphyte of this macroalga [36,37,38]. Annotation of the highly variable metabolites is often the main challenge to a full understanding of the biochemical processes involved in the detected changes. We therefore built molecular networks based on MS/MS data and GNPS, as this method has recently opened new avenues for the annotation of cyanobacterial metabolites [39,40,41].

## 2. Results

### 2.1. Major Metabolites from the Cyanobacterium Leibleinia gracilis

The freeze-dried cyanobacterium *L. gracilis* collected in the lagoon of Tahiti, French Polynesia, was extracted with a mixture of MeOH/DCM (1:1). A desalting fractionation process was performed by reversed phase vacuum liquid chromatography using a gradient elution of H_2_O, then MeOH, and then CH_2_Cl_2_. The fractions were analyzed by LC-MS, and the major metabolites of fraction F2 (H_2_O/MeOH 1:1) were then purified by reversed phase high performance liquid chromatography (HPLC), the other fractions showing only primary metabolites.

**Figure 1:**
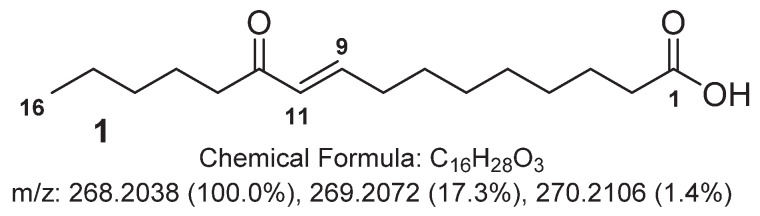
Structure of 11-oxopalmitelaidic acid (**1**).

The major compound **1** in the ELSD chromatogram of F2 was characterized by a HRMS spectrum with a protonated ion at *m*/*z* 269.2126 corresponding to a molecular formula C_16_H_28_O_3_ for the molecule (See Supplementary Material). Inspection of the ^1^H NMR spectrum evidenced a modified fatty acid derivative with characteristic signals of saturated methylenes at *δ*_H_ 1.30 and a methyl at *δ*_H_ 0.90 (t, *J* = 7.0 Hz, H_3_-16) (Figure 1, Table 1). Additionally, a conjugated olefin was inferred from signals at *δ*_H_ 6.91 (dt, *J* = 16.0, 7.0 Hz, H-9) and *δ*_H_ 6.11 (d, *J* = 16.0, H-10). The ^13^C NMR spectrum revealed the presence of a ketone with a characteristic signal at *δ*_C_ 203.8. HMBC correlations between the olefinic signals and this carbonyl moiety placed the double bond conjugated to the ketone. The location of this conjugated system was deduced from key H-13/C-11 and H-12/C-11 correlations following the assignment of signals corresponding to H-13 and C-13 through COSY, HSQC and HMBC correlations from the methyl C-16. The ^3^*J*_H9/H10_ coupling constant value of 16 Hz was indicative of an *E* configuration for the olefin, and therefore, **1** was named 11-oxopalmitelaidic acid.

### 2.2. Comparative Metabolomics Study 

**Figure 2:**
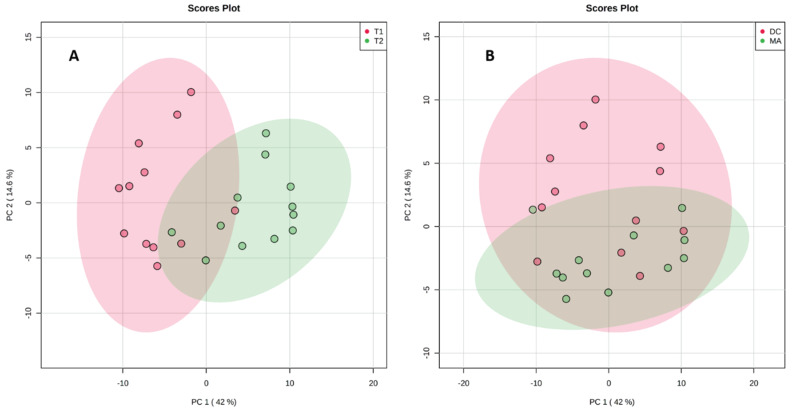
Principal component analysis (PCA) on the metabolomic profiles of all samples (**A**) before (T1) and after the rainy event (T2) and (**B**) according to the substrate macroalga (MA) or dead corals (DC).

A metabolomics approach was then applied to study the variability in the metabolome before and after a change of environmental conditions but also depending on the substrate of this cyanobacterium. A total of 24 samples (4 conditions, 6 biological replicates) collected before and after a rainy event (codes T1 and T2, respectively) both on the macroalga *Turbinaria ornata* and the dead corals (codes MA for macroalga and DC for dead corals) were analyzed from the same site in the lagoon of Tahiti. After filtration and normalization of the data obtained by UHPLC-HRMS, a total of 180 features were listed from the 24 samples. We first used an unsupervised principal component analysis (PCA) to study chemical variability according to the change of environmental conditions (Figure 2A) and then the substrate of the cyanobacterium (Figure 2B).

The results of the PCA analyses evidenced a better separation of the two groups in the situation 2A following a change in environmental condition. The impact of the substrate on the change in the metabolomic profiles is clearly lower, as shown by a large overlap between the two groups in Figure 2B. The PCA also demonstrated a large chemical variability along PC1 before and after the rainy event, with 42.9% variability and almost no change according to PC2 (Figure 2A). The situation is different for the influence of the substrate on the metabolomic profile (Figure 2B). In this case, the separation is not observed along PC1, and PC2 explains 14.6% of the global variability.

**Figure 3:**
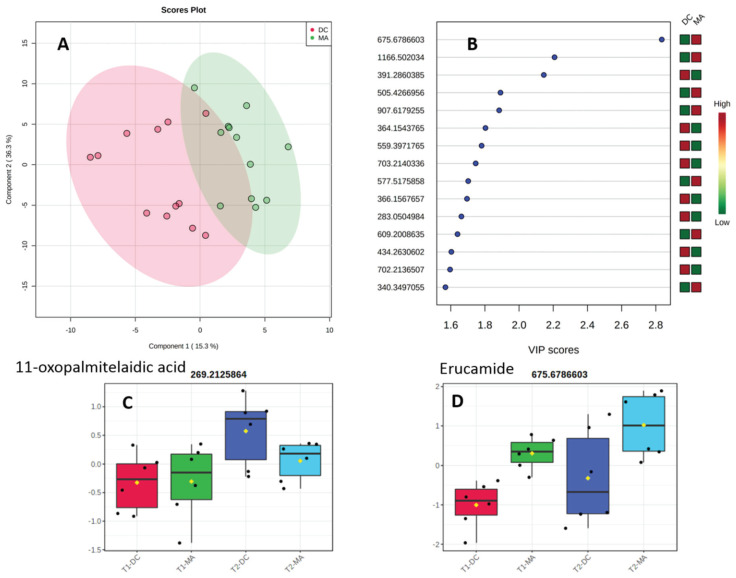
(**A**) Partial least-squares-discriminant analysis (PLS-DA) of the metabolome of the cyanobacteria on macroalga (MA) or dead corals (DC). (**B**) Variable importance in the PLS-DA (VIP) responsible for the discrimination and their scores. (**C**) Relative area of 11-oxopalmitelaidic acid (*m*/*z* 269.2126) in the four conditions. (**D**) Relative area of erucamide (*mz* 675.6787) in the four conditions.

Turning our interest toward the influence of the substrate on the metabolomic profiles of the cyanobacteria, we decided to assess the significance of the results obtained in the latter case. A supervised PLS-DA was therefore obtained and statistically analyzed (Figure 3A). The critical model parameter R^2^ was found to be higher than 0.6 using only one component, and the accuracy was found to always be higher than 0.7, revealing the good predictability of the model. The separation was also found to be statistically significant using a permutation test (1000) with a *p* value of 0.033. The significance of this result demonstrated an influence of the substrate on the metabolome of the cyanobacterium *L. gracilis* independent of the change in environmental conditions studied. The 15 variable importance in the PLS-DA (VIP) of highest value were then listed in order to highlight any family of metabolites over- or under-expressed with a change of substrate (Figure 3B). We were not able to identify the isolated metabolite 11-oxopalmitaleidic acid as a VIP, but its area was higher after the rainy event and on the dead coral (Figure 3C). For the annotation of the VIP, we first decided to search in databases of metabolites from cyanobacteria recently made available as the CyanoMet mass list [42] or the CyanoMetDB [43]. Unfortunately, no match was found in these two databases of metabolites. The VIP of highest score 2.8 was putatively assigned to (*Z*)-docos-13-enamide also known as erucylamide/erucamide with *m*/*z* 675.6787 and found as a dimer of *m*/*z* 338.3449 (Figure 3B) using the XCMS online Metlin database [44]. Interestingly, this compound is largely used as a slip agent in polymer science and may originate from contamination, but we cannot rule out a natural origin [45]. When looking at its relative area in the four different conditions, a higher concentration was observed on the macroalga and after the rainy event (Figure 3D). Most of the other VIP were found as minor metabolites and even if molecular formula could be proposed with confidence their annotation could not be performed. Some annotations were tentatively performed for the VIP at *m*/*z* 505.4267 with a molecular formula [2 × C_16_H_28_O_2_ + H]^+^ that could correspond to the dimer of a fatty acid in C16 containing two unsaturations. This proposition was also supported by the presence of the new fatty acid discovered as a major compound in the metabolome of this species. The other VIP at *m*/*z* 559.3971 provided a molecular formula of [2 × C_16_H_28_O_3_ + Na]^+^ corresponding to the sodium adduct of the dimer of the previously isolated compound (9*E*)-11-oxopalmitoleic acid (**1**). Interestingly, the latter compound was overexpressed on dead coral, while the less oxidized compound was more present on the macroalgal substrate (Figure 3B). 

**Figure 4:**
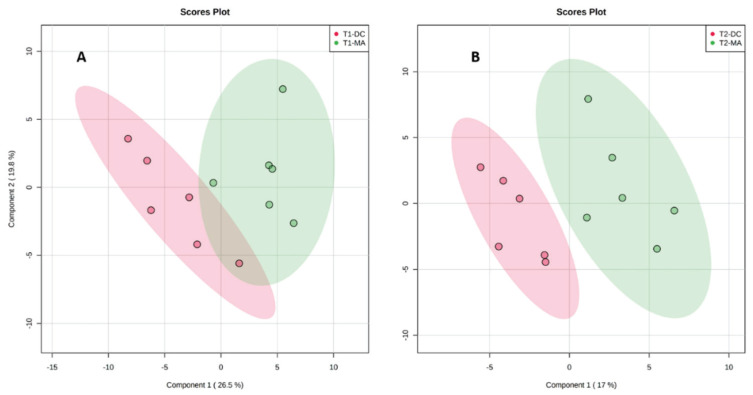
Partial least-squares-discriminant analysis (PLS-DA) for (**A**) samples before the rainy event (T1) and (**B**) samples after the rainy event (T2) in both substrates (MA and DC).

Following the preliminary observation of a strong influence of environmental conditions on the metabolome of the cyanobacterium, we then decided to run two separate statistical analyses on the samples before (T1) and after the rainy event (T2), focusing only on the variability according to the substrates in these two distinct environmental conditions. Two supervised PLS-DA were then obtained for both conditions (Figure 4). The separation between the two groups was clear for both analyses but more pronounced after the rainy event (Figure 4B). This was mainly explained by a change in the area of erucamide on macroalgae after the rainy event.

### 2.3. Molecular Networking

**Figure 5:**
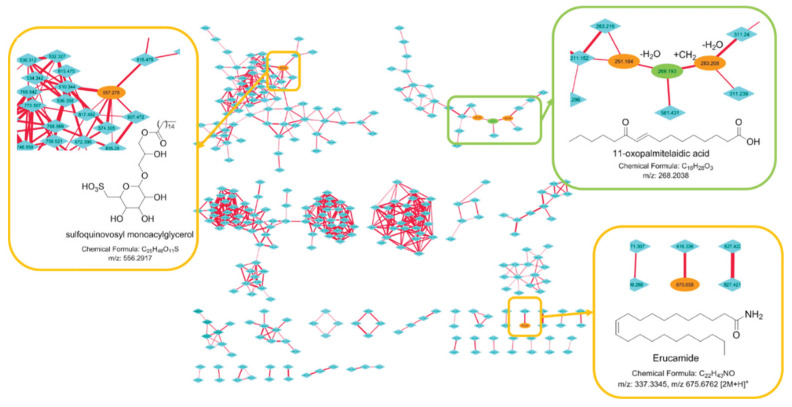
LC-HRMS/MS molecular network of the cyanobacterium *Leibleinia* *gracilis* built using GNPS with cosine similarity score cutoff 0.65. In green, the compound is isolated, and in orange, the compounds are assigned using databases or analogy.

The use of fragmentation patterns obtained by LC-HRMS/MS has appeared as a powerful tool to aid in dereplication processes to quickly annotate known metabolites using databases of MS/MS spectra [46]. HRMS/MS data are also at the basis of molecular networks that enable the visualization of structurally closely related metabolites grouped in clusters sharing similar fragments. In this study, we then performed an LC-HRMS/MS study of the methanol fraction of the cyanobacterium in the four conditions and built a molecular network to support the identification of the major metabolites. To this end, we used the natural products platform Global Natural Product Social Molecular Networking (GNPS) and the databases present to visualize the molecular network of the metabolome of this species (Figure 5) [47]. The major compound 11-oxopalmitelaidic acid isolated in the preliminary study was found in a cluster of fatty acid derivatives together with its dehydrated fragment at *m*/*z* 251.2 and a homologue at *m*/*z* 283.2. Erucamide in C_18_ was found in a small cluster of two compounds confirming its artefactual origin. Finally, the largest cluster in terms of nodes was identified as a cluster of glycoglycerolipid derivatives after the node at *m*/*z* 557.2 was annotated in the database of natural products of GNPS and Marinlit as a known sulfoquinovosyl monoacylglycerol. Two other large clusters of closely related metabolites could not be annotated through Marinlit, a database of marine natural products.

## 3. Discussion

The specialized metabolome of the studied benthic cyanobacteria *Leibleinia gracilis* collected in the lagoon of Tahiti was found to be composed of a very limited number of major metabolites (see SI for the HPLC profile). The main metabolite was purified and identified by NMR and MS as an oxylipin derivative, the uncommon 11-oxopalmitelaidic acid. This compound has only been reported once from the leaves of the plant *Ziziphus jujuba* but without NMR data provided, and there is no report so far of its presence in the marine environment [48]. Fatty acids are commonly found in cyanobacteria and C14, C16, and C18 derivatives have been identified as key chemical markers enabling the distribution of species into five main groups depending on their fatty acid profiles [49]. In the case of *L. gracilis*, fatty acids in C16 do seem favored, and while the unsaturation at C-9 is quite common for cyanobacterial fatty acids, the conjugated ketone at C-11 is rarely found. The molecular network built upon the metabolome of this species highlights a small cluster of derivatives in C16 produced in low quantity. Some modified fatty acids have also been isolated as major metabolites from species of other benthic cyanobacteria. For instance, the allenic acid puna’auic acid in C18 was also found as a major constituent of the metabolome of the cyanobacterium *Pseudanabaena* sp. collected in French Polynesia [50]. This fatty acid is also characterized by an unsaturation at C-9 and further oxidations at higher positions. The lyngbyoic acid in C14 featuring an uncommon cyclopropane moiety was identified as the major metabolite of a Caribbean species of *Lyngbya* cf. *majuscula* [51]. Interestingly, this major metabolite exhibited effects on the quorum sensing of the pathogenic bacteria *Pseudomonas aeruginosa* through the downregulation of a pigment biosynthesis but also of the expression of genes involved in quorum sensing. Even though we did not demonstrate this effect with 11-oxopalmitelaidic acid, the major fatty acid of the studied species *L. gracilis*, we can hypothesize similar activities for this metabolite, allowing the species to thrive in a highly competitive microenvironment. 

Metabolomics has proven to be a useful tool to assess the variability of the metabolome of cyanobacteria but it has mainly been applied using GC-MS approaches dedicated to the analysis of the primary metabolites [52]. More recently, metabolomic approaches coupled to genomic data were demonstrated as a promising tool to describe the specialized metabolome of cyanobacteria in depth [11,53,54,55]. The specialized metabolome of cyanobacteria is also known to be very variable with changes in environmental parameters, but studies using LC-MS metabolomic approaches are scarce and are mostly restricted to cyanobacteria in culture [56]. Weather conditions strongly affect the environmental parameters in coral reefs, and we therefore studied the change in the specialized metabolome of *L. gracilis* before and after a rainy event. The variability of the metabolome between these two conditions was found to be significantly higher than the variability induced by the substrate as some samples were found overgrowing dead corals but also the widespread macroalga *Turbinaria ornata*. At a lower extent, the substrate significantly influenced the metabolomic composition of the cyanobacterium revealing again the metabolomic plasticity of these microorganisms. Among the 15 most important VIP of the PLS-DA analysis, none could be annotated using the recently developed cyanobacterial databases of more than 2000 specialized metabolites [42,43]. This negative result points out the importance to build more extensive databases of cyanobacterial metabolites that should include fatty acid derivatives. Searching in other databases of metabolites, the main chemical marker of the substrate was found to be a fatty acid amide named erucamide in C_22_. This compound is largely used as a slip agent present in several polymers and may therefore have an anthropic origin [45], even though a natural origin cannot be totally rule out as cyanobacteria have been shown to contain fatty acid amides but usually largely modified and with shorter alkyl chain [57]. Its presence could be related to an adsorption on the surface of the macroalga as it is highly concentrated when *L. gracilis* is found as epiphyte of the macroalga. Macroalgae are known to accumulate organic matter at their surface and this organic matter may transfer the contaminants to the microorganisms found as epibionts. If we consider an unnatural origin, this result highlights the care that must be taken when using metabolomic studies to assess the variability of the metabolome in a species in the field as chemical markers might not be produced by the studied organisms but rather present as environmental contaminants. We finally used HRMS/MS spectra of the metabolites present in the cyanobacterium to help in the annotation of the VIP but also to visualize the clusters of closely related compounds present using the platform GNPS [47]. The molecular network built from the methanol fraction contained 33 clusters composed of 2–74 nodes. Glycoglycerolipids were potentially annotated to the main cluster while other large clusters where left non-annotated. This lack of result highlights the need for the construction of databases of MS/MS spectra of marine natural products including also primary metabolites and especially lipids that seem to represent a large percentage of the metabolites present.

## 4. Materials and Methods 

### 4.1. Study Site

**Figure 6:**
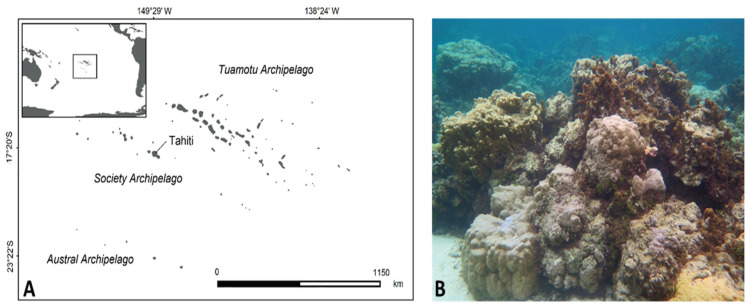
Localization of Tahiti Island (**A**) and illustrations of habitat (**B**).

The study took place in the lagoon of Tahiti, the main island of the Society Archipelago in French Polynesia (17,64402° S, 149,61430° W; Figure 6A). The climate in this region is characterized by two seasons—a dry and cold season from April to October with trade winds, and a hot and rainy season from November to March. The western side of the island is rimmed by a barrier reef system, with, in some locations, a deep channel and fringing structures that are heavily impacted by human activities. The sampling was performed during the hot and rainy season in the barrier reef of Punaauia near the reef crest in the same location on 11 and 19 February 2016, respectively. Heavy rains occurred on 14 February 2016 during a few days so the environmental conditions in the barrier reef have strongly changed between these two collections. The collection site is characterized by a habitat composed of dense compact coral heads (Figure 6B) and a very strong hydrodynamism. The floral assemblages were characterized by *Turbinaria ornata*, *Sargassum pacificum*, *Padina boryana*, *Dictyota bartayresiana*, and *Halimeda* spp. 

### 4.2. Biological Material

**Figure 7:**
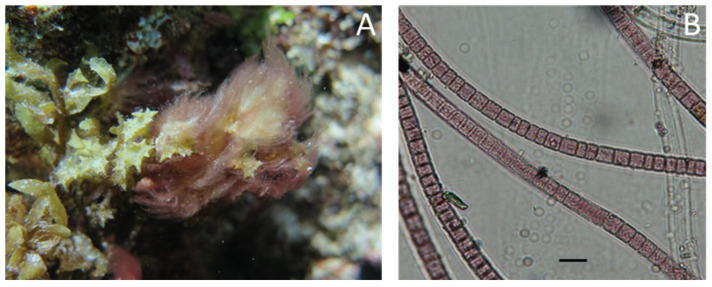
*Leibleinia gracilis* illustrations. (**A**) Tufts of *L. gracilis* overgrowing on the brown algae *Turbinaria ornata*. (**B**) Light microscopic images showing cell arrangements with fine constrictions at the cross-walls.

*Leibleinia gracilis* (Rabenhorst ex Gomont) Anagnostidis and Komárek 1988 (Figure 7A) is a cyanobacterium (Oscillatoriales, Oscillatoriaceae) commonly found in tropical waters and largely distributed in the Pacific Ocean. Thallus are pink, tufty, dense, floccose, and mucilaginous, generally up to 2 cm high (some specimens reach more than 10 cm). Trichomes are rose colored or pinkish, slightly constricted at cross-walls, 5–8 μm wide, not attenuated at the apex (Figure 7B). Cells are isodiametric or shorter than wide, usually 5 μm long with small granules within the plasma. Sheaths are thin, firm, smooth, and colorless. A representative specimen was sequenced (method described in Zubia et al. [22]), and the barcoding results are stored on GenBank under the accession number MT406770.

### 4.3. Sampling

The biomass and the replicates for metabolomics of *L. gracilis* were collected by snorkeling in the barrier reef of Punaauia (Figure 6A). Once harvested, the biomass was stored in plastic bags and placed on ice for transport to the laboratory. In the laboratory, the cyanobacteria were washed with demineralized water to remove epiphytes, epifauna, sand and other detritus and stored at −20 °C. In the laboratory, the monospecificity of each sample was checked under microscope. Then, the cyanobacteria were washed with demineralized water to remove epiphytes, epifauna, sand and other detritus and stored at −20 °C. Cyanobacteria were then freeze-dried and ground into powder before extraction. 

Six replicates of the cyanobacteria were collected in the same site before and after a rainy event, and they were all separated by a minimum of 2 m. Before and after the rainy event (codes T1 and T2, respectively), cyanobacteria were collected both on the macroalga and the dead corals (codes MA for macroalga and DC for dead coral). For each of the four sets of samples, the replicates were collected, washed, and stored on ice immediately in the field. For each sample, a subsample was preserved in a solution of buffered formaldehyde in seawater (3%) for microscopic examination to verify the identity of *L. gracilis*. The biomass of the remaining cyanobacteria was then freeze-dried and ground into powder before extraction.

### 4.4. Chemical Diversity of L. gracilis

A freeze-dried biomass of the cyanobacterium *L. gracilis* (36 g) collected in the same site was extracted with a mixture of MeOH/DCM (1:1, three times 250 mL) under sonication. The resulting 5.7 g extract was fractionated by C18 reversed-phase vacuum liquid chromatography into five fractions after elution with a gradient of H_2_O-MeOH from 100:0 to 0:100 and MeOH/DCM (50:50) (*v*/*v*). Fractions were analyzed by NMR (500 MHz, Agilent, Santa Clara, CA, USA) and analytical HPLC using an Agilent 1260 Infinity HPLC equipped with DAD and ELSD detectors. Purification of the fraction F2 (H_2_O/MeOH 1:1) was performed by reversed phase semi-preparative HPLC (Symmetry, C18 column, 100 Å, 7 µm, 7.8 × 300 mm), with a gradient of solvent A: H_2_O + 0.1%TFA, solvent B: CH_3_CN + 0.1%TFA, 0–3 min: 20%B, 3–30 min: 100%B. Compound **1** was therefore isolated as the major compound with a mass of 3.5 mg. Structure elucidation was performed by NMR (500 MHz, Agilent) and high-resolution mass spectrometry on an UHPLC-qToF (Agilent 6540 ).

### 4.5. Comparative Metabolomics and Molecular Metwork

#### 4.5.1. Sample Preparation and Analyses

A biomass of approximately 500 mg of freeze-dried material per replicate of *L. gracilis* was extracted three times with a mixture DCM/MeOH (1:1) under sonication. Solid phase extraction of the extracts obtained after evaporation of the solvents was carried out using 1 g C18 cartridges with three consecutive eluents: H_2_O, MeOH, and DCM (two cartridge volumes each). After evaporation of the MeOH fractions under vacuum, the fractions were dissolved in 500 µL of MeOH and filtered on a 0.2 µm PTFE filter prior to metabolomics analyses.

Analyses were carried out on an Agilent 1290 UHPLC equipped with a pump, auto sampler, and UV diode array detector and coupled to a high-resolution Agilent 6540 Q-ToF mass spectrometer equipped with a dual AJS ESI source. Mass spectra were acquired in positive mode from *m*/*z* 100 to 1500 for all samples at a scan rate of 3 spectra/s. This mass range enabled the presence of two reference mass ions including purine (C_5_H_4_N_4_ at *m*/*z* 121.0509) and hexakis(1H, 1H, 3H-tetrafluropentoxy)phosphazene (C_18_H_18_O_6_N_3_P_3_F_24_ at *m*/*z* 922.0098). The elution rate was set at 0.35 mL·min^−1^, and the injection volume 3 *μ*L. LC-MS grade chromatographic solvents were composed of A: water with 0.1% formic acid (FA) and B: acetonitrile 0.1% formic acid. Elution was performed on a reversed phase pentafluorophenyl column (Waters, 1.7 μm, 130 Å, 100 × 2.1 mm) maintained at 40 °C and using the following gradient: 20% CH_3_CN (+ 0.1% FA) during 2 min; from 20% CH_3_CN (+ 0.1% FA) to 100% CH_3_CN (+ 0.1% FA) in 14 min; hold at 100% CH_3_CN (+ 0.1% FA) for 2 min; and back to the initial conditions and equilibration for the last 4 min. The quality control (pool) was prepared by mixing 50 µL of all 24 replicates. MS parameters were set for positive mode as follows: capillary voltage 3500 V, nebulizer pressure 35 psi, drying gas 12.0 L.min^−1^, gas temperature 300 °C, SheathGasTemp 300 °C, SheathGas 8.0 L·min^−1^, fragmentor 175 V, skimmer1 65.0 V, octopole RF Peak 750 V, vaporizer 200 V, voltage charge 2,000 V.

#### 4.5.2. Data Analyses for the Metabolomic Study

Raw datasets from UHPLC-HRMS were first converted to .mzML files using the MSConvert software. These files were then treated by XCMS under R version 3.3.3 software. XCMS parameters were used as follows: peak picking (method = “centwave”, ppm = 15, peakwidth = c (5, 20), snthresh = 1000, retention time correction (method = “obiwarp”), matching peaks across the samples (bw = 2, minfrac = 0.5, mzwid = 0.015). The data were combined into a single matrix by aligning with the peak intensity, *m*/*z* values and retention times. The matrix was filtered according to coefficient of variation (CV) of the features calculated on pool samples (CV > 25%) were removed and missing values removed manually. A total of 180 features were identified between all specimens studied. Data analyses were performed using the MetaboAnalyst online software [58]. Log transformation and pareto scaling were used for normalization. Observation of the results was realized using PLS-DA statistical analysis. Permutational multivariate analysis was also performed to identify significant factors linked to metabolites diversity. Raw data are stored on the MetaboLights platform under the accession number MTBLS1462 [59]

#### 4.5.3. Data Analyses for the Molecular network

MS/MS raw data were converted into an .mzXML file using the MSConvert software [60]. The Molecular Network was constructed on the GNPS webserver [47] using the following parameters: cosine threshold set at 0.65 value, precursor mass tolerance of 2.0 Da, fragment mass tolerance of 0.5 Da. Cystoscape 3.6.1 [61] was used to visualize network. The node color represents the source of the precursor ion, and the edge thickness illustrates the interpretation of the cosine similarity score. Sub networks were created to show details of node connectivity. Annotation was performed manually using the MarinLit database after creating a molecular network.

## 5. Conclusions

The metabolome of the cyanobacterium *L. gracilis* blooming in the lagoon of Tahiti is dominated by fatty acids/amides that might have important ecological functions. An uncommon oxidized fatty acid was found to be the major metabolite of this species, and its concentration slightly increased after a rainy event. Using an untargeted metabolomic study, we could assess that the main influence on the metabolome is due to environmental factors. A lower variability was related to the substrate of the benthic cyanobacterium. Only few metabolites could be annotated that shows how critical is the construction of cyanobacterial metabolites databases including lipid derivatives and not only toxins. The main VIP was identified as erucamide, a slip agent largely used in polymers, highly present when the cyanobacterium was collected as epiphyte of the macroalga *Turbinaria ornata*.

## Figures and Tables

**Table 1:** ^13^C (150 MHz) and ^1^H (600 MHz) NMR data of compound **1** in CD_3_OD.

Position	*δ*_C,_ mult.	*δ*_H_, mult. (*J* in Hz)
1	177.8, qC	-
2	34.8, CH_2_	2.29, t (7.0)
3	25.8, CH_2_	1.61, m
4	29.9, CH_2_	1.38, m
5	30.0, CH_2_	1.31, m
6	29.9, CH_2_	1.38, m
7	29.0, CH_2_	1.51, quint (7.0)
8	33.4, CH_2_	2.26, q (7.0)
9	149.7, CH	6.91, dt (16.0, 7.0)
10	131.3, CH	6.11, d (16.0)
11	203.8, qC	-
12	40.6, CH_2_	2.59, t (7.0)
13	25.5, CH_2_	1.58, m
14	32.8, CH_2_	1.31, m
15	23.5, CH_2_	1.32, m
16	14.3, CH_3_	0.91, t (7.0)

## Supplementary Materials

HPLC-ELSD-DAD and UHPLC-HRMS chromatograms of the fraction F2. NMR data of 11-oxopalmitelaidic acid. The following are available online at https://www.mdpi.com/2218-1989/10/5/215/s1.
