# Supplementary material for: Insights into the Metabolome of the Cyanobacterium Leibleinia gracilis from the Lagoon of Tahiti and First Inspection of Its Variability
Source: Metabolites. 2020 May 24;10(5):215. doi: 10.3390/metabo10050215 (PMC7281704; doi:10.3390/metabo10050215)
Supplement: Supplementary file 1 [file metabolites-10-00215-s001.pdf]

Insights into in the metabolome of the cyanobacterium Leibleinia gracilis from the lagoon of Tahiti and first inspection of its variability

Hiren Solanki[1], Manon Pierdet[2], Olivier P. Thomas[1,*], and Mayalen Zubia[2,*]

[1] Marine Biodiscovery, School of Chemistry and Ryan Institute, National University of Ireland Galway, University Road, H91 TK33 Galway, Ireland
[2] UMR Ecosystèmes Insulaires Océaniens, LabEx CORAIL, University of French Polynesia, Faa'a, BP6570, 98702 Tahiti, French Polynesia

P2 Figure S1. Analytical HPLC chromatograms of the F2 fraction of *L. gracilis* on C18 reverse phase (UV at 210 nm and ELSD)

Figure S2. UHPLC-HRMS chromatogram and MS/MS fragmentation of the isolated major (9*E*)-11-oxopalmitoleic acid

P3 Figure S3. ^{1}H NMR of (9*E*)-11-oxopalmitoleic acid in CD_3OD (600 MHz)

Figure S4. ^{13}C NMR spectrum of (9*E*)-11-oxopalmitoleic acid in CD_3OD (150 MHz)

P4 Figure S5. COSY spectrum of (9*E*)-11-oxopalmitoleic acid in CD3OD (600 MHz)

Figure S6. HSQC NMR of (9*E*)-11-oxopalmitoleic acid in CD3OD (600 MHz)

P5 Figure S7. HMBC NMR of (9*E*)-11-oxopalmitoleic acid in CD3OD (600 MHz)

P6 Figure S8. UHPLC-HRMS chromatogram showing the MS/MS fragmentation spectrum of the main annotated compound Erucamide (*m/z* 338.3449)

Figure S9. MS/MS fragmentation of the Erucamide: confirmation matching with Metlin database

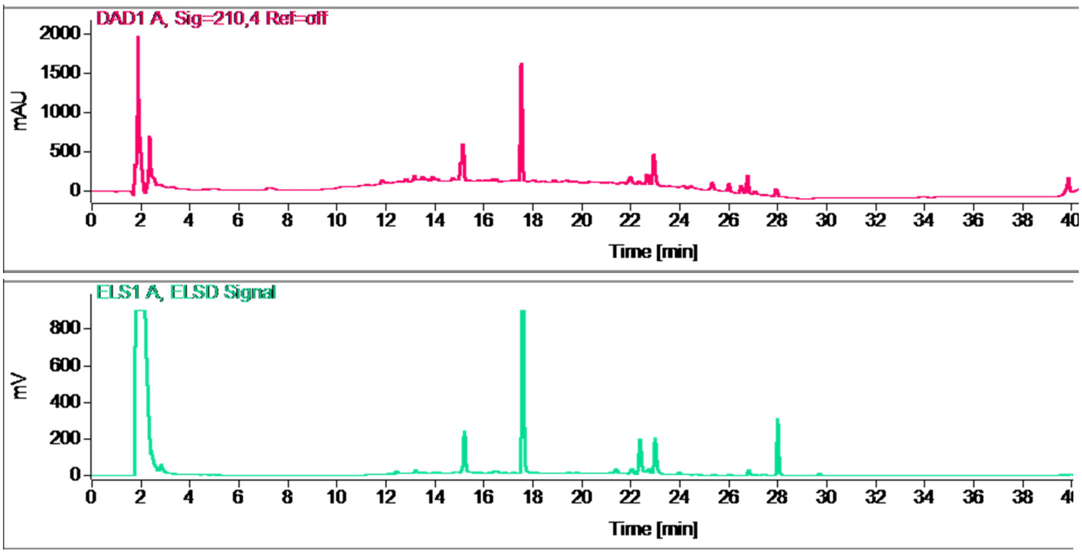

Figure S1. Analytical HPLC chromatograms of the F2 fraction of *L. gracilis* on C18 reverse phase (UV at 210 nm and ELSD)

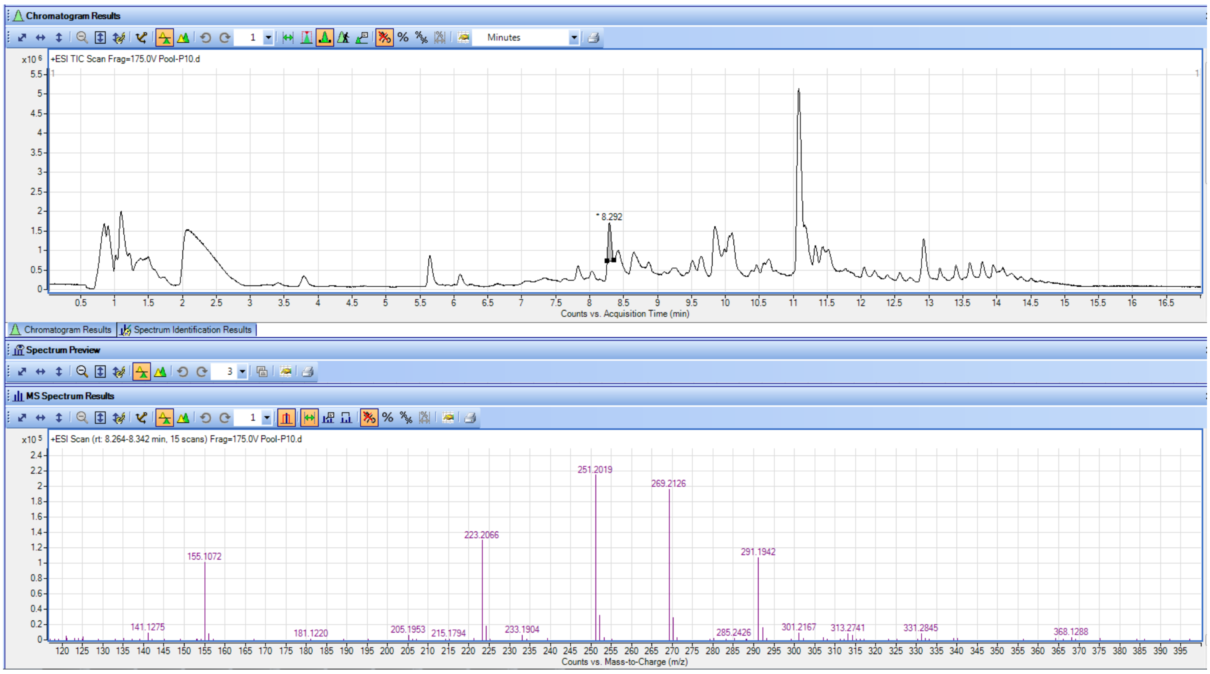

Figure S2. UHPLC-HRMS chromatogram and MS/MS fragmentation of the isolated major (9*E*)-11-oxopalmitoleic acid

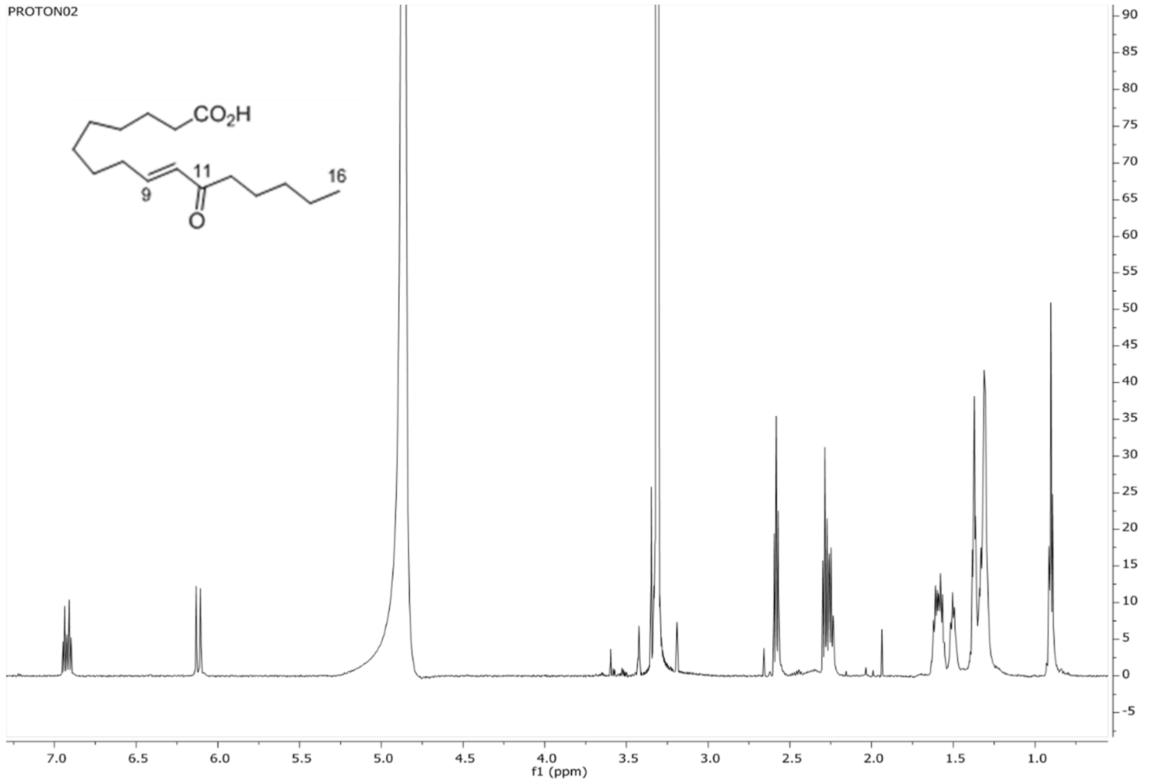

Figure S3. ^{1}H NMR of (9*E*)-11-oxopalmitoleic acid in CD_3OD (600 MHz)

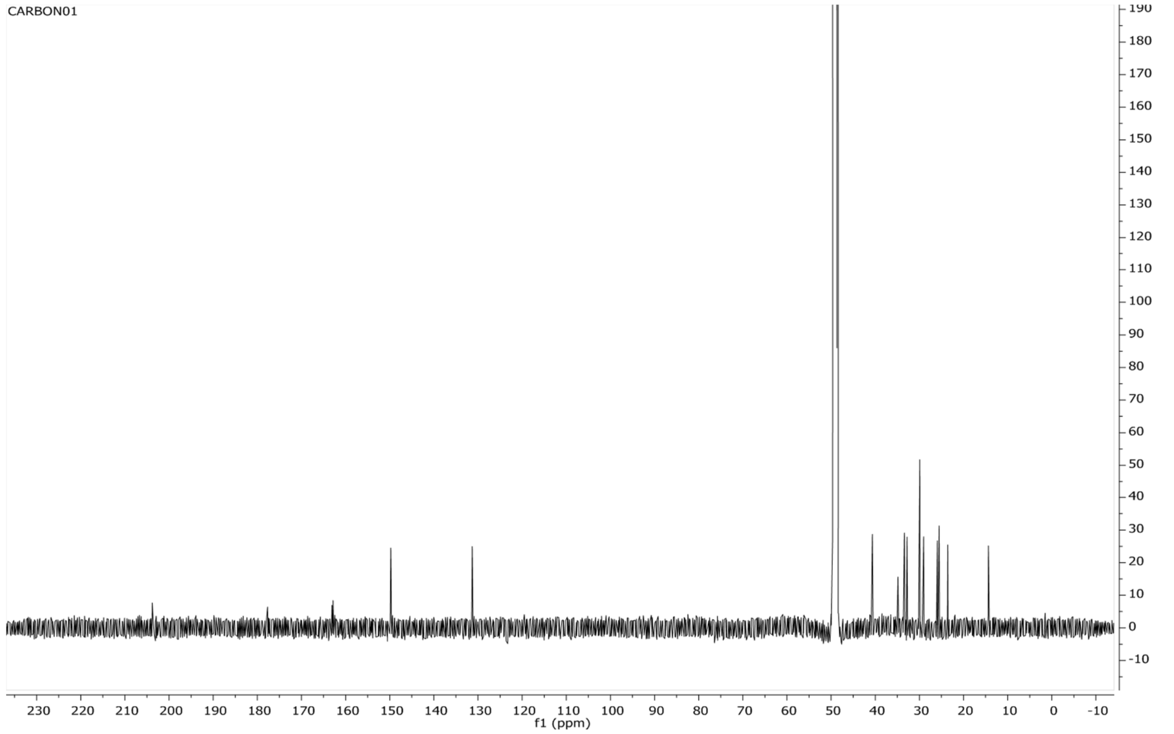

Figure S4. ^{13}C NMR spectrum of (9*E*)-11-oxopalmitoleic acid in CD_3OD (150 MHz)

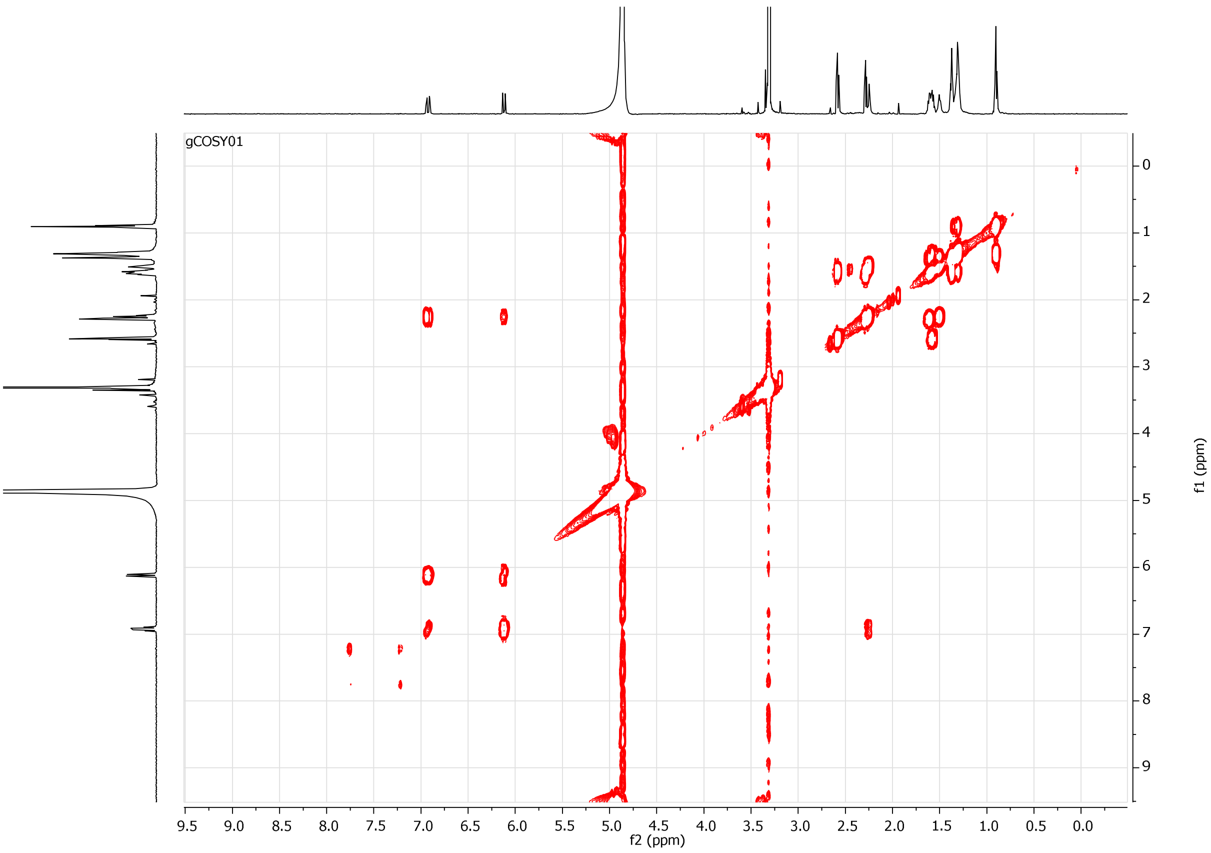

Figure S5. COSY spectrum of (9*E*)-11-oxopalmitoleic acid in CD_3OD (600 MHz)

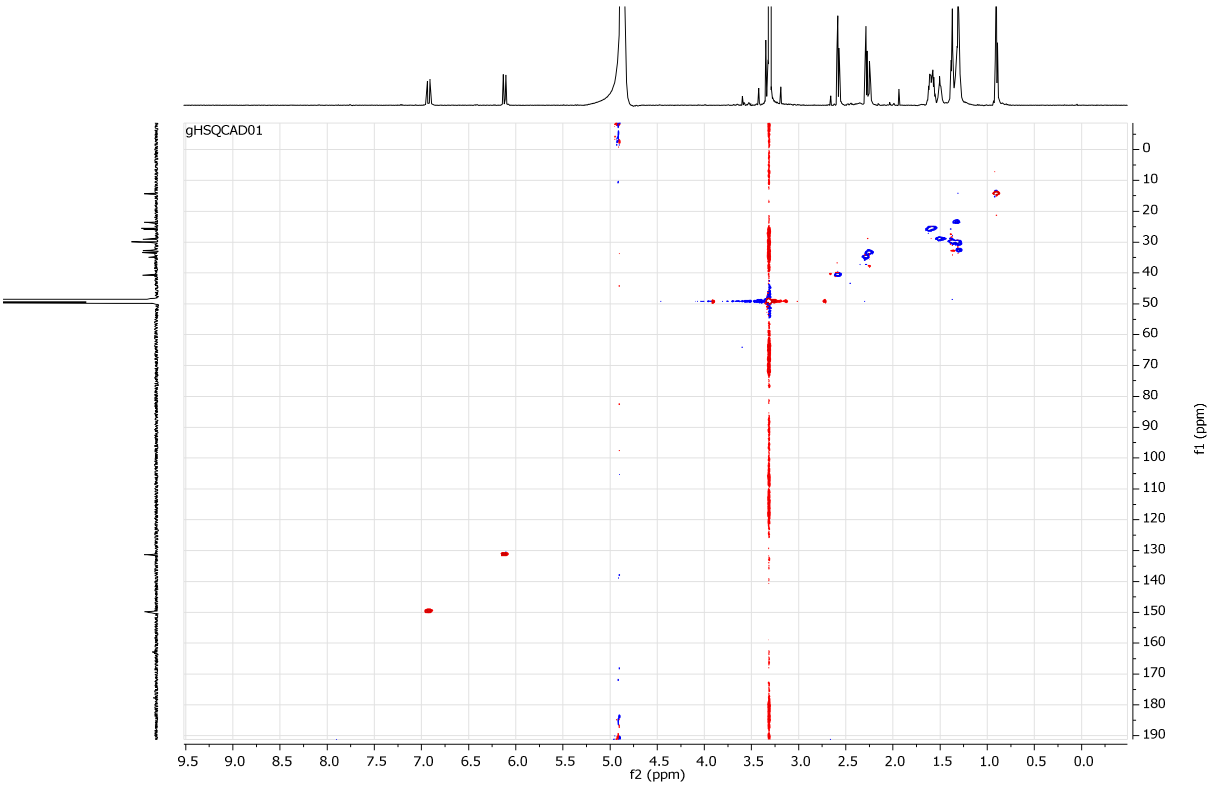

Figure S6. HSQC NMR of (9*E*)-11-oxopalmitoleic acid in CD_3OD (600 MHz)

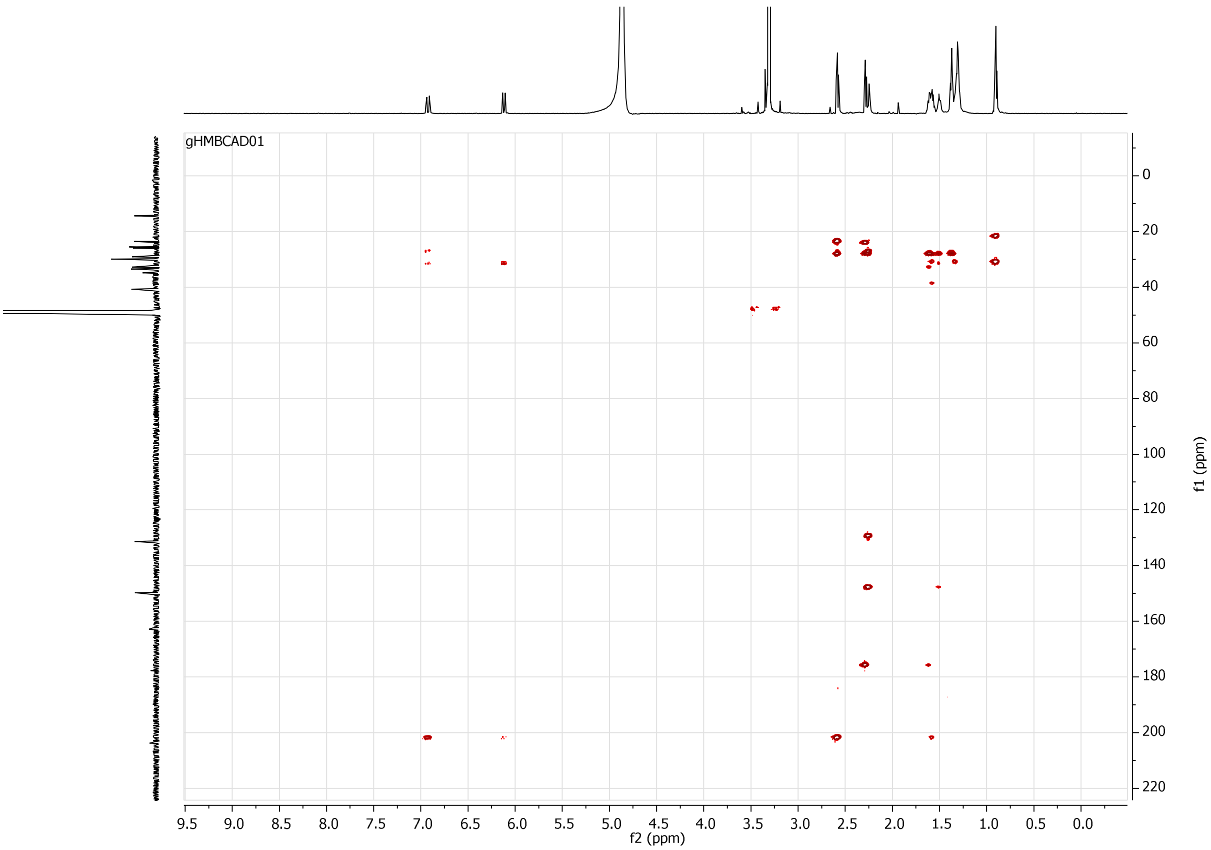

Figure S7. HMBC NMR of (9*E*)-11-oxopalmitoleic acid in CD_3OD (600 MHz)

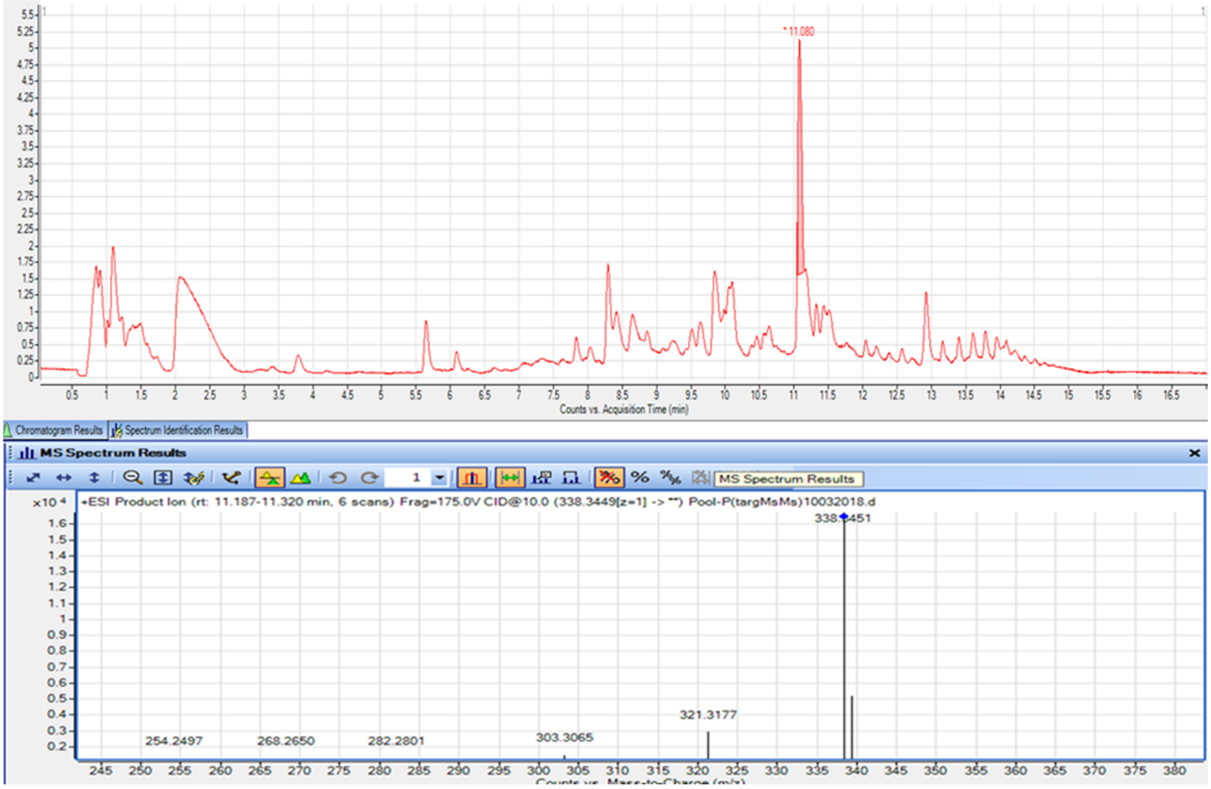

Figure S8. UHPLC-HRMS chromatogram showing the MS/MS fragmentation spectrum of the main annotated compound Erucamide (*m/z* 338.3449)

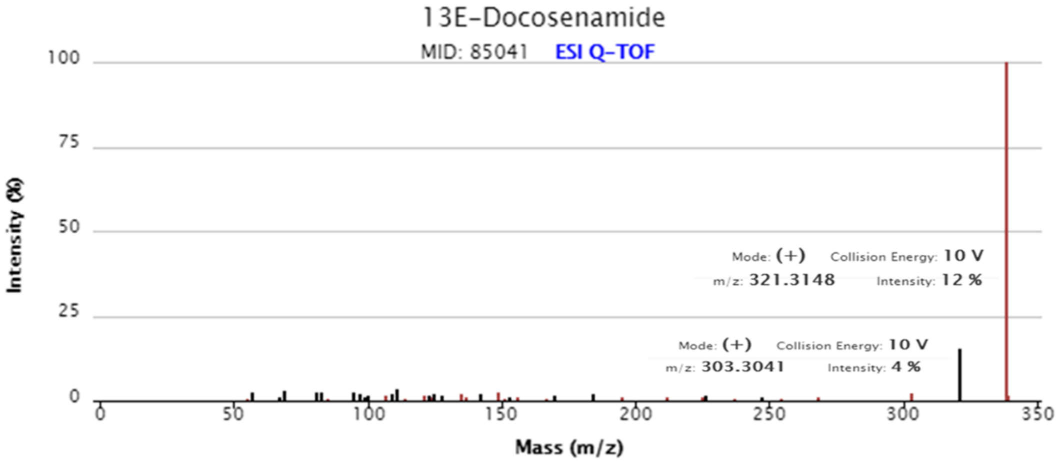

Figure S9. MS/MS fragmentation of the Erucamide: confirmation matching with Metlin database
